# Erratum: Chen, Y.-R., et al. Improvement of Impaired Motor Functions by Human Dental Exfoliated Deciduous Teeth Stem Cell-Derived Factors in a Rat Model of Parkinson’s Disease. *Int. J. Mol. Sci.* 2020, *21*, 3807

**DOI:** 10.3390/ijms22031154

**Published:** 2021-01-25

**Authors:** Yong-Ren Chen, Pei-Lun Lai, Yueh Chien, Po-Hui Lee, Ying-Hsiu Lai, Hsin-I Ma, Chia-Yang Shiau, Kuo-Chuan Wang

**Affiliations:** 1 Division of Neurosurgery, Department of Surgery, National Taiwan University Hospital, Taipei 100, Taiwan; tefu.chen@caire.com.tw; 2 Graduate Institute of Medical Sciences, National Defense Medical Center, Taipei 114, Taiwan; 3 Non-Invasive Cancer Therapy Research Institute, Taipei 104, Taiwan; pohuilee@outlook.com; 4 Genomics Research Center, Academia Sinica, Taipei 11529, Taiwan; d01b48001@ntu.edu.tw; 5 Cancer Progression Research Center, National Yang-Ming University, Taipei 11221, Taiwan; g39005005@gmail.com; 6 Department of Medical Research, Taipei Veterans General Hospital, Taipei 11217, Taiwan; d49405004@gmail.com; 7 Department of Neurosurgery, Tri-Service General Hospital, Taipei 115, Taiwan; uf004693@mail2000.com.tw; 8 Department of Surgery, National Defense Medical Center, Taipei 115, Taiwan; 9 Graduate Institute of Life Sciences, National Defense Medical Center, Taipei 114, Taiwan

The authors regret to have made a mistake in publishing this paper [1], with incorrect information on experimental protocols. 

In Section 4.3. on page 12, the first sentence “All experimental protocols were approved by Human Subject Research Ethics, National Defense Medical College (Taipei, Taiwan) (ID: IACUC-17-083, 21 March 2017)” should be corrected to “The collection of human deciduous teeth was approved by the Institutional Review Board of Tri-Service General Hospital (Taipei, Taiwan) (TSGH IRB, No.: 2-108-05-103, 21 March 2019)”. The change does not affect the scientific results.

The authors would like to apologize for any inconvenience caused to the readers by this change.
