# Supplementary material for: Improvement of Impaired Motor Functions by Human Dental Exfoliated Deciduous Teeth Stem Cell-Derived Factors in a Rat Model of Parkinson’s Disease
Source: Int J Mol Sci. 2020 May 27;21(11):3807. doi: 10.3390/ijms21113807 (PMC7312764; doi:10.3390/ijms21113807)
Supplement: Supplementary file 1 [file ijms-21-03807-s001.pdf]

Supplementary Materials

Improvement of Impaired Motor Functions by Human Dental Exfoliated Deciduous Teeth Stem Cell-Derived Factors in a Rat Model of Parkinson's Disease

Yong-Ren Chen[1,2,3], Pei-Lun Lai[4], Yueh Chien[5,6], Po-Hui Lee[3], Ying-Hsiu Lai[6], Hsin-I Ma[7,8], Chia-Yang Shiau[2,9†*], Kuo-Chuan Wang[1†*]

[1] Division of Neurosurgery, Department of surgery, National Taiwan University Hospital, Taipei 100, Taiwan. tefu.chen@caire.com.tw (Y.-R. C.); wang081466@yahoo.com.tw (K.-C. W.)
[2] Graduate Institute of Medical Sciences, National Defense Medical Center, Taipei 114, Taiwan. tefu.chen@caire.com.tw (Y.-R. C.); hehcys1234@gmail.com (C.-Y. S.)
[3] Non-invasive Cancer Therapy Research Institute - Taiwan, Taipei 104, Taiwan. pohuilee@outlook.com (P.-H. L.); tefu.chen@caire.com.tw (Y.-R. C.)
[4] Genomics Research Center, Academia Sinica, Taipei 11529, Taiwan. d01b48001@ntu.edu.tw (P.-L. L.)
[5] Cancer Progression Research Center, National Yang-Ming University, Taipei 11221, Taiwan; g39005005@gmail.com (Y. C.)
[6] Department of Medical Research, Taipei Veterans General Hospital, Taipei 11217, Taiwan. g39005005@gmail.com (Y.C.); d49405004@gmail.com (Y.-H. L.)
[7] Department of Neurosurgery, Tri-Service General Hospital, Taipei 115, Taiwan. uf004693@mail2000.com.tw (H.-I. M.)
[8] Department of Surgery, National Defense Medical Center, Taipei 115, Taiwan. uf004693@mail2000.com.tw (H.-I. M.)
[9] Graduate Institute of Life Sciences, National Defense Medical Center, Taipei 114, Taiwan. hehcys1234@gmail.com (C.-Y. S.)

* Correspondence: wang081466@yahoo.com.tw (K.-C. W.); hehcys1234@gmail.com (C.-Y. S.); Tel: +886-25171167; Fax: +886-25171129.
[†] These authors contributed equally to this work.

Figure S1

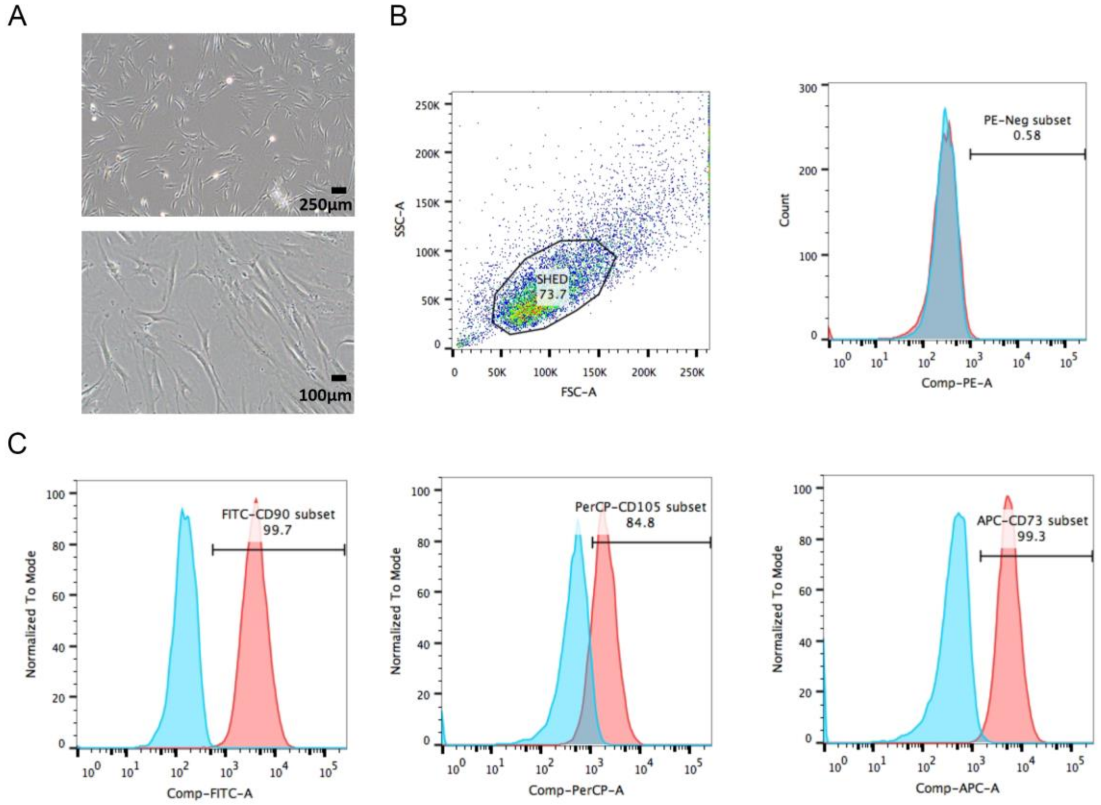

Figure 1. Characterization of the mesenchymal features of SHED. (A) Microscopic examination of SHED at passage 3 shows the fibroblast-like morphology of SHED after cultivation. (B) Flow cytometry analysis shows that SHED are negative for hematopoietic markers, CD34 and CD45, (C) and are positive for MSC markers, CD73, CD90, and CD105.
